# Primary Medical Schools, Medical Reforms

**Published:** 1851-11

**Authors:** 


*Primary Medical Schools, Medical Reforms.*—An announcement of the opening of a primary medical school at Knoxville, east Tennesee, under the charge of Frank H. Ramsay, M. D., has been received. We have noticed, of late, advertisements of others who propose to devote a portion of their time to systematic private instruction.

The establishment of schools of this description is one of the measures of reform recommended by the *American Medical Association.* Dr. Ramsay, in his announcement, remarks as follows:—

"The discussions of the subject of Medical Education, by the members of the Association, have produced the impression, that though in some particulars, the chartered Medical Colleges may be improved,—yet the great root of defective qualifications in those whom they diplomate, is the want of ability to appropriate facilities which are intended for students well grounded in the rudiments of medical acquirements. And it is believed that this inability proceeds wholly from inefficient primary instruction, resulting, not from incompetency on the part of private instructors, but from disinclination, growing out of an absence of that peculiar stimulant to inclination and energy, pecuniary interest."

These remarks are just. That students leaving medical schools, do not exhibit the degree of proficiency which is to be desired, arises, in not a small number of instances, from their deficiency in rudimentary acquirements, and in mental discipline, without which they can but imperfectly profit by collegiate advantages. No one measure of reform would tend so much to raise the scientific character of the medical profession, as to secure, in some way, a higher standard of intellect and attainment in those commencing the study of medicine.

Next to this, in importance, are progressive improvements in medical instruction at medical colleges. Such improvements, there is every reason to believe, are going on. At no former period, perhaps, were greater energy and efficiency displayed than at the present time, in the medical institutions of our country. This springs, in a considerable degree, from the spirit of emulation excited by the multiplication of medical schools. It is common, we know, to attribute to the increased number of schools, evil consequences. The effect, doubtless, is to diminish the claims of superiority arrogated by institutions depending for continued success on the *prestige* of the past. A monopoly in this, as in every other branch of industry, would doubtless conduce to the pecuniary profit of those interested in preventing competition. But, so far as the welfare of medical science and education is concerned, we can see no good reason why a fair and honorable rivalry should not be beneficial. In evidence of the fruits of a multiplication of schools, in this point of view, let any one compare the methods and amount of teaching, as now pursued, with the state of medical instruction twenty years ago. No one qualified to make this comparison will deny that the medical sciences are more fully and better taught, in proportion to the present condition of knowledge, than was the case formerly. To what is this owing, if not to the emulation incident to competition?

Next in order to progressive improvements in medical colleges, we would rank *private instruction.* Here has been a sad deficiency in the means of medical instruction. A large proportion of medical students receive no instruction whatever, except while attending medical lectures. Their nominal preceptors take no interest in their studies, save to supply them with a few works which may have become obsolete, and to inculcate some *practical* routine rules which, possibly, are, at a future day, to be unlearned. Here is, as Dr. Ramsay remarks, a great root of defective qualifications in those whom they (the schools,) diplomate. To obviate this drawback on medical education, private or summer schools are highly desirable, designed to fill up, profitably, the time not spent in attending lectures, by a regulated system of study, with proper explanations, illustrations, and examinations. After a proper preliminary qualification before entering on the study of medicine, the advantages just mentioned, are, at the present time, most needed.

On this subject, the following extract from a letter by Prof. Daniel Drake, addressed to Dr. Ramsay, is pertinent. Prof. D. writes strongly, but perhaps not more so than the merits of the matter require. He says:

"The egregious fallacy, that young men may become good physicians and surgeons, by *listening* to a couple of courses of lectures, will, I hope, soon be as obvious to every body as it has long been to myself. We say a great deal about the want of preliminary education in literature, and the shortness of our medical terms, but observation has convinced me, that the greatest of all drawbacks, is the want of protracted, systematic etymological study in standard works, aided by catechetical examinations, and required manuscript compositions on the existing topics of study — that is, on the very subjects the student is investigating. I firmly believe that this is the best foundation to build upon; and that very few students who rely, as, unfortunately is now the case, throughout the United States, on lectures only, will ever become eminent men. They go the University, after a few months of desultory reading,—while there they *cannot* read, and during the next summer they *do not;* for, having been subjected to the external stimuli of the lecture room, solitary book study is insipid and irksome.

A private school, such as you contemplate, should have a small laboratory in which some first lessons of chemistry could be taken, and an autopsic apartment, in which the student might work; and after thus pursuing his studies for at least two years, he might attend public lectures, without the danger of being intellectually ruined, by making himself, for a couple of winters, the *passive* receptacle of the outpourings of half a dozen lecturers; which outpourings mix together but never unite: He goes away with a full stomach, but an empty thoracic duct."

Finally, a fourth measure of medical reform, is not to be lost sight of, which, in importance, is hardly second to either of those mentioned. We allude to constant efforts of personal improvement on the part of members of the medical profession — in other words, adopting the expression of our friend, Prof. Coventry, in an excellent address on this subject, published in a former volume of this Journal, *self reformation.* The importance of this measure appears to be overlooked by many who are constantly harping on the deterioration of the medical profession. Medicine is emphatically progressive. A few years of sloth suffices for a medical practitioner to fall far behind the increasing requirements of the science. Yet, how many take little or no pains to keep pace with the progress of medical knowledge? These stationary practitioners are like dead weights upon the profession. They injure the character of the profession, not only by their own retrograde position, but by holding others back. Too indolent to have any concern for personal improvement, they are not satisfied to have others advance; hence they oppose all measures designed for the benefit of their neighbors, as well as themselves, and too often is it the case that jealousy and conscious inferiority lead to the exhibition of a malevolent spirit which has furnished occasion for the reproach so often uttered, that members of the medical profession cannot live on terms of amity and mutual respect. It cannot, and it should not be concealed that if the medical profession, as a profession, be obnoxious to charges affecting its character both in a scientific and a moral point of view, the fault is not to be laid at the door of medical schools, nor attributed to aught extrinsic to the profession itself. The *status* of the profession is to be elevated by the profession. Who else can do it? And just as high a position as it may acquire for itself, may it be expected will be preserved by those who are constantly entering its ranks. If it be asked why young physicians are not more uniformly proficients in the recent improvements in medical science, we answer, not because these improvements are not adequately taught at medical schools, but because the student finds that they are not deemed necessary by those already in successful practice. Take, for example, physical exploration. There are few institutions, probably, in which this branch is not taught, yet how little is it, even now, in use with practitioners. Why is it so? Simply because the lessons of the public teachers do not receive co-operation or encouragement out of the lecture room, and may even be derided by those by whose attainments and success the student limits his own aspirations.

As in scientific attainment, so in moral excellence, the candidate for admission into the profession is apt to measure his aims by what he sees already to exist in the profession. If he finds detraction, backbiting, and ignoble tricks to be in accordance with usage, it can hardly be expected that he will cultivate honorable sentiments; and, hence, to secure worthiness of character in the profession *which is to be*, the profession *which is* must exemplify virtues for imitation.

